# Breast Adenocarcinoma and Cold Agglutinin Disease: A Paraneoplastic Syndrome

**DOI:** 10.7759/cureus.74437

**Published:** 2024-11-25

**Authors:** Christopher W Honore, Upasana Agrawal, Faryal Shoaib, Shivani Sharma

**Affiliations:** 1 Department of Internal Medicine, Louisiana State University Health Sciences Center, Shreveport, USA; 2 Department of Pathology, Louisiana State University Health Sciences Center, Shreveport, USA; 3 Department of Oncology, Medical University of South Carolina, Charleston, USA

**Keywords:** cold agglutinins, immune hemolytic anemia, metastatic breast disease, primary breast malignancy, therapeutic plasmapheresis

## Abstract

Autoimmune hemolytic anemia is a disorder that is characterized by the destruction of red blood cells through an autoimmune process, such as temperature-dependent antibodies. The two predominant types, cold agglutinin and warm agglutinin disease, typically possess different underlying etiologies. Prompt recognition and workup of autoimmune hemolytic anemia should be prioritized to potentially uncover any underlying primary cause, such as malignancy. Here, we present the case of a 50-year-old female who presented with new-onset facial numbness and altered vision, in addition to fatigue and jaundice. Serum studies revealed evidence of severe hemolytic anemia, and subsequent imaging and biopsy confirmed the presence of metastatic breast adenocarcinoma. Breast adenocarcinoma is the most common cancer diagnosed in women in the United States, and it can be associated with paraneoplastic syndromes such as humoral hypercalcemia of malignancy. For breast cancer, autoimmune hemolytic anemia is a rare associated finding, especially as a presenting sign of malignancy. Therapy for her hemolysis and malignancy began promptly, which included starting anti-hormonal therapy for her malignancy and several courses of prednisone, which prompted moderate improvement. Plasmapheresis was initiated following a decline that showed short-term improvement to bridge to a more comprehensive cancer therapy that she ultimately was unable to tolerate. While autoimmune hemolytic anemia is rarely secondary to solid tumors, clinicians should maintain a high index of suspicion to uncover and treat serious illnesses.

## Introduction

Autoimmune hemolytic anemia (AIHA) is a form of hemolytic anemia that is driven by an autoimmune process, usually antibodies that activate the complement system. It is typically seen in lymphoproliferative malignancies and is seldom seen in solid tumors for reasons that are poorly understood. In addition, cold agglutinin disease is relatively uncommon in AIHA secondary to malignancy, as these manifestations are usually of the warm agglutinin type. Both types can lead to severe, clinical manifestations of hemolysis. AIHA can be an example of a paraneoplastic syndrome, which is a clinical syndrome that results from a malignancy stimulating an immune response or releasing certain substances; therefore, these syndromes can be a presenting sign of cancer. Recognizing that AIHA can be a presenting sign of malignancy can encourage a prompt workup to potentially uncover any underlying malignancy. We, therefore, report the case of a 50-year-old female whose initial workup was significant for cold agglutinin disease that was resistant to steroids but improved following plasmapheresis. She was diagnosed with metastatic breast carcinoma upon further workup and started therapy.

## Case presentation

A 50-year-old female with a history of human immunodeficiency virus (HIV), noncompliant with antiretroviral therapy, presented with new-onset right-sided facial numbness and blurry vision that had progressed over a day before presentation. She endorsed fatigue, shortness of breath, poor appetite, and weight loss of more than 10 pounds over the past month. She was a current smoker with a 16-pack-year smoking history. Her mother had a history of breast cancer. Physical examination showed a temperature of 98 Fº (36.7 Cº), a blood pressure of 103/59 mmHg, a pulse of 90 BPM, a respiratory rate of 22 breaths per minute, and an oxygen saturation of 100% on room air. She had dry mucous membranes, jaundiced skin, and bilateral lower leg edema. She had hepatomegaly. A complete neurological examination showed decreased sensation to the right side of the face in a distribution of V1, V2, and V3 without weakness or evidence of cranial nerve (CN) VII palsy. Her right pupil was pinpoint, whereas the left pupil was 4 mm and reactive.

Laboratory studies were obtained that greatly supported hemolytic anemia due to increased lactate dehydrogenase (LDH) and indirect bilirubin (explaining the patient's jaundice) and a markedly decreased hemoglobin (Table 1). While her reticulocytes were elevated, her reticulocyte index was 0.65; this, in conjunction with her hemolysis labs, suggests that while she is hemolyzing, she is simultaneously underproducing RBCs; this suggests marrow involvement. The peripheral smear showed spherocytes (classically seen in AIHA), evidence of moderate agglutination, anisocytosis, and rare schistocytes. The patient had a positive direct antiglobulin test (RBCs coated with complement), supporting autoimmune hemolysis. This is further supported by an elevated IgM cold agglutinin titer. The antinuclear antibody was negative. Her HIV could have been the etiology of her autoimmune hemolysis as she was not taking her antiretroviral medication; however, a normal CD4 count ruled out this possibility. These laboratory findings in conjunction with her clinical findings of fatigue, hepatomegaly, and jaundice strongly suggested autoimmune hemolytic anemia, possibly due to an underlying malignancy in the setting of marrow underproduction and new-onset neurologic symptoms. This prompted urgent imaging.

**Table 1:** Laboratory studies

Laboratory Studies	Value	Reference Range
Hemoglobin	3.6 g/dL	10.9 - 14.3 g/dL
White blood cells	17.33 K/uL	3.90 - 12.70 K/uL
Platelets	83 K/uL	150 - 450 K/uL
Aspartate aminotransferase	119 U/L	10 - 40 U/L
Alanine aminotransferase	72 U/L	10 - 44 U/L
Total bilirubin	5.1 mg/dL	0.1 - 1.0 mg/dL
Direct bilirubin	3.1 mg/dL	0.1 - 0.3 mg/dL
Reticulocyte count	5.60%	0.5 - 2.5%
Ferritin	2630 ng/mL	20.0 - 300.0 ng/mL
Vitamin B12	>2000 pg/mL	210 - 950 pg/mL
Folate	16.6 ng/mL	4.0 - 24.0 ng/mL
Haptoglobin	114 mg/dL	30 - 250 mg/dL
Lactate dehydrogenase	340 U/L	110 - 260 U/L
IgM cold agglutinin titer	128 titer	< 64 titer
CD4 cell count	1082/mm^3^	401 - 1532/mm^3^

**Figure 1:**
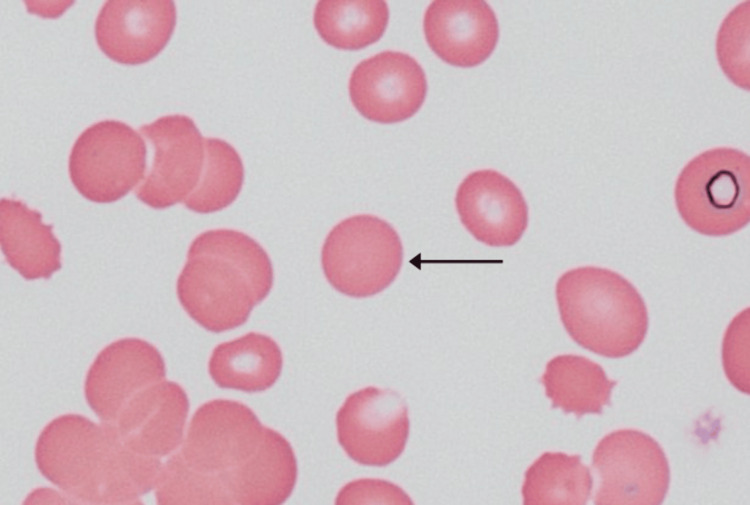
Bone marrow aspirate smear Spherocyte (black arrow): Spherocytes are classically seen in either autoimmune hemolytic anemia (AIHA) or hereditary spherocytosis due to breakdown in the spleen from either antibody-mediated splenic recognition (AIHA) or structural deformities (hereditary spherocytosis).

**Figure 2:**
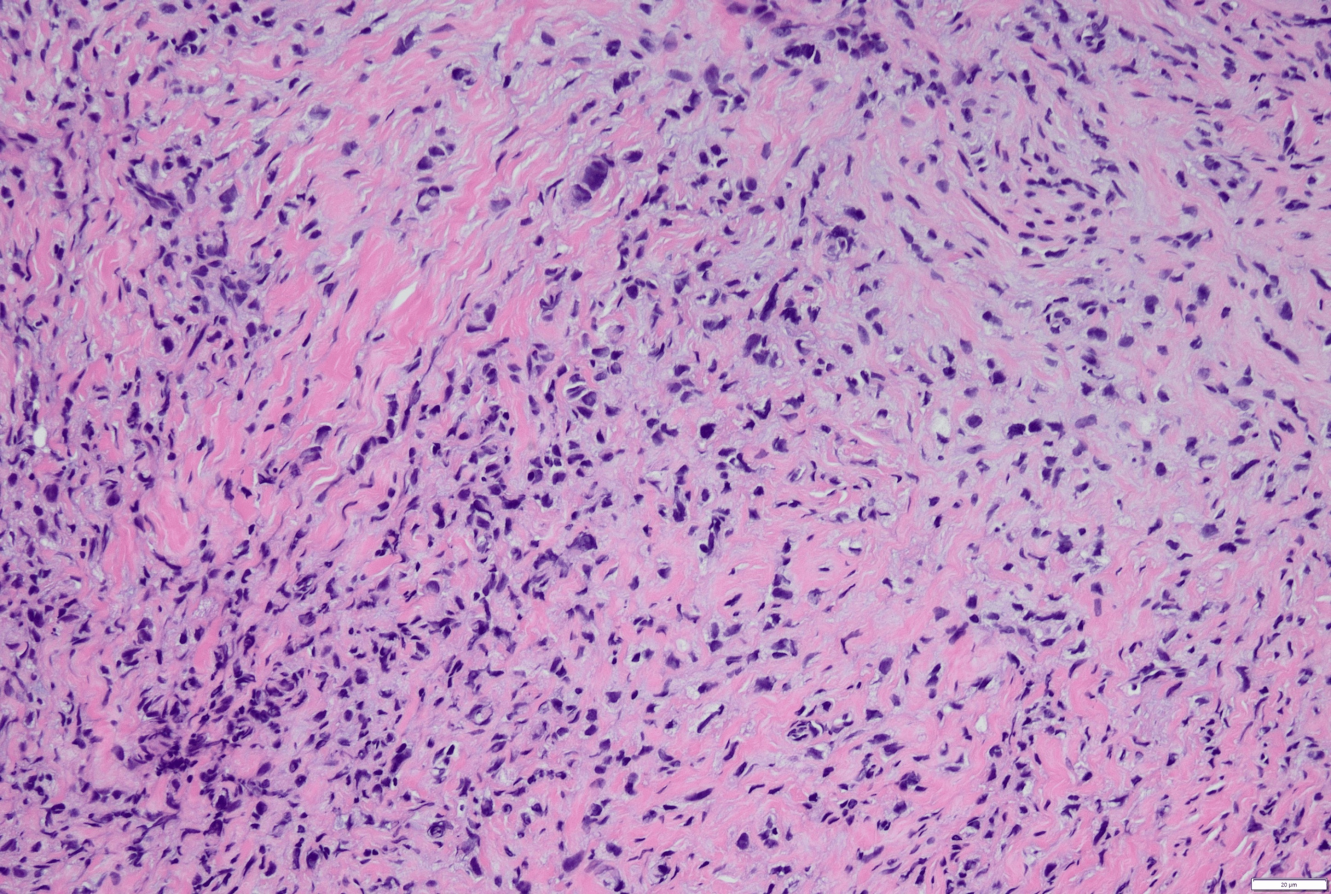
Core-needle biopsy breast specimen Hematoxylin and eosin staining of obtained tissue showing invasive lobular carcinoma of the breast.

CT of the chest, abdomen, and pelvis showed a right breast soft tissue density, in addition to diffuse osteosclerosis throughout the visualized skeleton. CT triple-phase showed a right hepatic mass with ring-like enhancement. MRI brain revealed diffuse nonspecific pachymeningeal enhancement accompanied by right inferior cerebellar hemisphere encephalomalacia and gliosis, indicative of metastatic disease that had led to her neurologic manifestations. Bone marrow biopsy was obtained to complete the workup for anemia and showed a hypocellular marrow with metastatic malignant cells, likely breast cancer. The bone marrow aspirate showed spherocytes (Figure 1). Lumbar puncture was performed with cerebrospinal fluid studies indicating metastatic breast disease. A core needle biopsy of the breast mass showed low-grade, estrogen receptor/ progesterone receptor (ER/PR)-positive, human epidermal growth factor receptor 2 (HER2)-negative invasive lobular carcinoma (Figure 2).

The patient received two units of packed red blood cells (PRBCs) transfusion in the emergency department for her severe anemia, with clinical improvement. She was started on anastrozole. Her hemoglobin continued to downtrend on hospital day four and was found to be 6.6 g/dL. Steroids were considered our strongest option at this time due to their literature evidence of efficacy. Prednisone 1 mg/kg was initiated on day seven of hospitalization and continued for four days. The patient’s hemoglobin increased to 8 g/dL, and cold agglutinin titers decreased, which corresponded with clinical improvement with fatigue and jaundice. Following the discontinuation of prednisone, her hemoglobin trended down to 6.6 g/dL on day 13 of hospitalization, increasing her fatigue. She was restarted on prednisone, which continued until she was discharged. During a follow-up oncology clinic appointment three weeks after her hospitalization, the patient was found to have a hemoglobin of 4.0 g/dL, which prompted readmission. During her second admission, she completed three rounds of plasmapheresis with clinical and laboratory improvement. Goals of care discussion was held with the patient and her family as her prognosis was guarded due to metastatic breast cancer. The patient opted to pursue palliative treatment and was initiated on ribociclib. She was unable to tolerate treatment, as it led to multiple episodes of anemia and thrombocytopenia requiring transfusions. Given her advanced metastatic disease and rapidly declining hemodynamic status, the patient and her family decided to pursue hospice care.

## Discussion

AIHA, especially cold agglutinin disease, is rarely reported in solid tumors. One study found that just 30% of all AIHAs associated with a solid tumor are of the cold agglutinin type [1]. What makes the association even more challenging to demonstrate is the timing when the hemolysis occurs. AIHA has been found to occur prior to the cancer diagnosis, during treatment, or after treatment. Puthenparambil et al. found that while AIHA has been seen in all solid cancers, it is more likely to be seen in renal cell carcinoma and Kaposi sarcoma [1]. A case report describes a similar presentation as our patient, who presented with AIHA secondary to underlying breast carcinoma [2]. As such, it is necessary to maintain a high index of suspicion regarding this diagnosis. Prompt recognition can lead to an early diagnosis of the underlying cause and initiation of therapy.

To potentially uncover any underlying cause of AIHA, it is paramount to recognize its cardinal signs and symptoms. As with any form of anemia, the patient can present with fatigue, shortness of breath, and pallor. Symptoms more specific for hemolytic anemia include jaundice, hepatosplenomegaly, flank pain, and kidney injury. Laboratory evaluation will show an elevated reticulocyte count, bilirubin, and LDH, and decreased haptoglobin. A peripheral blood smear showing spherocytes could also indicate autoimmune hemolytic anemia. A standard confirmatory test is a direct antiglobulin test, which demonstrates RBCs bound by antibodies. Further characterization of these antibodies can help determine the subtype of AIHA, as seen in this patient with elevated cold agglutinin titers. A unique component of cold agglutinin disease (CAD) is the effect of temperature. In CAD, symptoms are associated with fluctuations in temperature, and rapid cooling or cold temperatures can trigger hemolysis. The distal extremities are most at risk; therefore, acrocyanosis can be a predominant symptom [3]. Overt symptoms of hemolysis can also occur, as in our patients.

Therapy for cold agglutinin disease, which has no underlying cause, is usually focused on avoidance of cold triggers, especially of the distal extremities. In patients with less severe forms of the disease, this can be adequate to control hemolysis [4,5]. Transfusions can be administered safely as long as products are appropriately warmed using in-line blood warmer and by avoiding the usage of blood products with high plasma content to avoid inadvertent transfusion of extraneous complement protein that may exacerbate hemolysis [5,6]. For unresponsive disease, further therapy is indicated, with rituximab being an effective first-line therapy in conjunction with plasmapheresis and transfusions as needed [4,6]. Nearly all cold immunoglobulins are IgM, and as such, they are primarily intravascular and are efficiently removed from the vasculature using plasmapheresis. Unfortunately, there is no evidence-based treatment for cold agglutinin syndrome, or cold AIHA secondary to an underlying disease or malignancy, as all recommendations in the literature are based on case reports and clinical experience [6]. It stands to reason that resolution of the underlying condition will lead to improvement in symptoms. Achieving complete remission in some curable malignancies, such as aggressive lymphomas, can result in the resolution of hemolysis. While steroids are not recommended in the treatment of CAD as they only control hemolysis in just 15% of cases, there are differing opinions and practices regarding their use in cold agglutinin syndrome (CAS) [6,7]. In this case, our patient had some tangible improvement following the initiation of steroids, which supports the existing literature. There are similar cases where steroids were given, causing an improvement in symptoms [2,6,8]. If hemolysis is unrelenting, plasmapheresis has been found to be helpful, along with RBC transfusions with proper precautions [6]. These therapies should be focused on controlling hemolysis while attempting to correct the underlying infection or malignancy to achieve long-term resolution.

It is important to note that due to the rarity of this CAS secondary to a solid tumor, there is a relative lack of data in the literature. Cases may be underreported, and therefore clinical characteristics and prognosis of these patients are unclear.

## Conclusions

Cold agglutinin syndrome (CAS), a type of AIHA, involves circulating IgM antibodies that trigger hemolysis in the vasculature secondary to another disorder. If secondary to malignancy, it is often linked to lymphoproliferative disorders and rarely solid tumors. AIHA can develop before, during, or after cancer treatment. It is important for clinicians to recognize AIHA as a possible complication of solid tumors to facilitate prompt diagnosis and treatment. Future areas of study could analyze the efficacy of treatment modalities in CAD, but the rarity of such a presentation is severely limited. Our patient presented with hemolytic anemia that was secondary to an already metastatic breast cancer in several parts of the body, and as such her disease was severe and difficult to manage; earlier recognition is required. Paraneoplastic syndromes often complicate the diagnostic process and should therefore encourage a swift multidisciplinary approach to uncover and treat undetected malignancy.
